# Vitamin D deficiency was common among nursing home residents and associated with dementia: a cross sectional study of 545 Swedish nursing home residents

**DOI:** 10.1186/s12877-017-0622-1

**Published:** 2017-10-10

**Authors:** Rebeka Arnljots, Jörgen Thorn, Marie Elm, Michael Moore, Pär-Daniel Sundvall

**Affiliations:** 1 Närhälsan, Research and Development Primary Health Care, Region Västra Götaland, Research and Development Center Södra Älvsborg Sweden, Sven Eriksonsplatsen 4, SE-503 38 Borås, Sweden; 2 Department of Public Health and Community Medicine/Primary Health Care, Institute of Medicine, Sahlgrenska Academy at the University of Gothenburg, Box 454, SE-405 30 Gothenburg, Sweden; 3 Närhälsan Heimdal Health Care Center, Stengärdsgatan 22, SE-503 34 Borås, Sweden; 4 Head Nurse, Health Care Unit Borås Municipality, Ramnåsgatan 1, SE-501 80 Borås, Sweden; 5 Academic Unit of Primary Care and Population Sciences, Faculty of Medicine, University of Southampton, Aldermoor Health Centre, Aldermoor Close, Southampton, SO16 5ST UK; 6 Närhälsan Sandared Health Care Center, Strandvägen 11, SE-518 32 Sandared, Sweden

**Keywords:** Vitamin D, Homes for the aged, Nursing homes, Frail elderly, Dementia, Infectious disease, Antibiotics

## Abstract

**Background:**

Residents of nursing homes may have low 25-hydroxyvitamin D (25OHD) concentrations. Associations between vitamin D and cognitive performance, dementia and susceptibility to infections are not clearly established. The aim of this study was to investigate the prevalence of vitamin D deficiency and to identify associated factors among residents of nursing homes for elderly.

**Methods:**

In this cross-sectional study blood samples for analysis of 25OHD were collected from all participating residents of Swedish nursing homes for the elderly from January to March 2012.

Exclusion criteria: dementia too severe to collect a blood test, terminally ill or refusing participation. Outcome Measures: Serum 25OHD concentrations. Logistic regression to evaluate factors associated with vitamin D deficiency (25OHD < 25 nmol/L).

**Results:**

Blood samples were obtained from 545 of 901 residents of 22 nursing homes. Mean age 86 years (SD 6.9), 68% were women. Prevalence of vitamin D supplementation 17%, dementia 55%, lack of appetite ≥3 months 45% and any antibiotic treatment during the last 6 months 30%. Serum 25OHD concentrations: mean 34 nmol/L (SD 21, median 27, range 4–125), 82% (448/545) had 25OHD < 50 nmol/L and 41% (224/545) had 25OHD < 25 nmol/L. Adjusted OR (95% CI; *p*-value) for possible predictors of vitamin D deficiency (25OHD < 25 nmol/L): vitamin D supplementation 0.075 (0.031–0.18; *p* < 0.001), lack of appetite ≥3 months 0.75 (0.50–1.1; *p* = 0.15), hours outdoors/week 0.99 (0.96–1.0; *p* = 0.62), Fitzpatrick skin phototype (4–6) 0.69 (0.44–1.1; *p* = 0.12); dementia 2.3 (1.5–3.4; p < 0.001) and antibiotics last 6 months 1.6 (1.1–2.6; *p* < 0.029), adjusted for age and gender.

**Conclusions:**

Vitamin D deficiency was common among nursing home residents and strongly associated with dementia. Regardless of causality or not, it is important to be alert for vitamin D deficiency in nursing homes residents with dementia. As expected vitamin D supplementation was associated with less vitamin D deficiency, however lack of appetite, staying outdoors and skin phototype were not significant predictors. Antibiotic treatments during the last 6 months were associated with vitamin D deficiency, potentially supporting the hypothesis that vitamin D deficiency is associated with infections.

## Background

Vitamin D refers to a group of fat-soluble molecules, from which the most important in humans are vitamin D3 (cholecalciferol) and vitamin D2 (ergocalciferol) [1]. A small amount of these can be obtained from the diet, but about 90% comes from synthesis of vitamin D in the skin when exposed to ultraviolet B radiation from the sun [2, 3]. This dermal synthesis depends on the area of skin exposed to sunlight, duration of exposure, latitude, season and skin type [4, 5]. In Sweden, latitude 55–69° North, there is no dermal synthesis of vitamin D during the winter months [1, 4]. Vitamin D has a significant role in mineralization of bone, skeletal maturation, regulating the concentration of calcium and phosphate, therefore severe vitamin D deficiency can cause osteomalacia in adults and rickets in pediatric population [6]. Vitamin D deficiency is a known risk factor causing fractures, osteoporosis and muscle weakness in elderly [5, 7, 8].

Vitamin D deficiency might be associated with reduced cognitive functions and dementia, but further research is required regarding this [9–14]. Vitamin D receptors have been discovered throughout the body including the nervous system, the cardiovascular and endocrine system causing biological actions including inflammation and amyloid plaque formation in the brain, potentially related to cognitive decline and Alzheimer’s disease, and inhibition of vascular smooth muscles proliferation potentially related to cardiovascular disease [15]. Vitamin D also affects the immune system [16–20], therefore it is important to study if elderly residents of nursing homes with vitamin D deficiency also have more infections. Several studies during recent years have observed associations between vitamin D deficiency and several medical conditions such as cognitive decline, cancer, diabetes, cardiovascular mortality, autoimmune diseases, metabolic diseases, neurological diseases, lung diseases and increases mortality rate [3, 13–15, 21–27].

There is still a lack of consensus worldwide regarding the optimum serum concentrations of vitamin D [28–30]. A suggestion is to consider 25-hydroxyvitamin D (25OHD) < 12.5 nmol/L as severe deficiency, 12.5–25 nmol/L as moderate deficiency and 25–50 nmol/L as mild deficiency [31]. It was recommended during a meeting among European acknowledged experts in the field of vitamin D that elderly (>65 years) should have 25OHD ≥ 50 nmol/L [29] which corresponds with the Nordic Nutrition Recommendations [32]. Other studies suggest 25OHD ≥ 75 nmol/L as optimum serum concentration [33, 34]. Since there is debate over optimal concentrations of 25OHD we report prevalence of deficiency at a range of values and elected to use an arbitrary cut point of 25 for analysis of predictors.

Several international studies have shown low serum concentrations of vitamin D in elderly individuals and the concentrations have been even lower among those living in nursing homes and long term care facilities [19, 24, 29, 35–43]. Elderly people are at risk for lower concentrations of vitamin D as a result of decreased dietary intake, decreased cutaneous synthesis and less time spent outdoors [15]. A difference is expected in serum concentrations of vitamin D when comparing populations of different countries due to variations in fortification of food, tradition regarding use of supplements, the dominant skin type within the population, amount of sunlight exposure and nutritional status. There is one recently reported study regarding the prevalence of vitamin D deficiency among the elderly residents of Swedish nursing homes [24]. However, in this study the blood samples were collected during different seasons of the year and many of them were collected when the elderly moved into the nursing homes, thus expected to have higher serum concentrations of vitamin D. Therefore, further studies are required in Sweden regarding the prevalence of vitamin D deficiency among elderly residents of nursing homes. It is also unknown whether previously known risk factors for vitamin D deficiency such as less time spent outdoors, lack of appetite and dark skin color are significant risk factors also for elderly residents of Swedish nursing homes. There is a need for further research on possible associations between vitamin D deficiency and dementia and susceptibility to infections.

The aim of this study was to investigate the prevalence of vitamin D deficiency and to identify associated factors among residents of nursing homes for elderly.

## Methods

During the first 3 months of 2012, a case report form was completed and blood samples collected from all included residents of 22 nursing homes in south-western Sweden (latitude 57.58° North to 57.82° North). The attending nurses were provided detailed verbal and written information for the study procedure. The study was approved by the Regional ethical review board of Gothenburg University (D-nr 578–11). The data was collected along with another study of interleukin-6 concentrations in the urine and antimicrobial resistance in urinary pathogens among Swedish nursing home residents [44, 45].

### Inclusion and exclusion criteria

All residents of the participating nursing homes were invited to participate. Those accepting participation were included if they met the following inclusion criteria:

- Permanent residence in nursing homes for the elderly, regardless of gender and for how long time they have been residing there
- Presence at a nursing home for the elderly during the study
- Participation approval
- Residents with dementia were included if cooperative when collecting blood samples
- Not terminally ill

The following exclusion criterion was used:

- If the resident did not agree to participate or discontinued study participation

### Statement of consent

All residents were informed of the study both verbally and in writing. From decision-capable individuals choosing to participate in the study, informed approval was collected. However, a considerable number of participants consisted of residents with dementia of varying degrees. If the resident was incapable of understanding the given information and thereby possessing a reduced decision capability, these residents only participated so long as they both: Did not oppose participation in the study and that the appointed representatives or relatives did not oppose their participation after being provided with information about the study. The Regional ethical review board of Gothenburg University approved this procedure.

### Case report form

In addition to collecting the blood sample, the attending nurse made an entry in the case report form for each included resident regarding: age, gender, vitamin D supplementation or not, lack of appetite in the last 3 months, diagnosis of dementia in the medical record and if the resident had received any antibiotic treatment during the last 6 months as a marker of potential bacterial infection. The diagnosis of dementia in the medical records required a previous comprehensive history and physical examination by a physician, laboratory tests, cognitive function test and most often neuroimaging. The average number of hours outdoors per week during April to August was registered, this is the period during which the sun exposure at our northern latitude is sufficient to induce the vitamin D synthesis in the skin. Regarding skin phototype, a simplified Fitzpatrick skin phototype [46] was used; Fitzpatrick skin phototype 1–3 was registered as lighter skin phototype and 4–6 was registered as darker skin phototype.

### Laboratory tests

The attending nurse collected blood samples from each participating resident. The blood samples were analysed at the laboratory of clinical chemistry at the Södra Älvsborg Hospital in Borås, Sweden, using routine clinical procedure. The blood samples were chilled before transport and usually arrived at the laboratory within 24 h. Measurements of the concentrations of 25OHD in serum were analyzed by the LIAISON® 25 OH Vitamin D TOTAL Assay (DiaSorin Inc., Stillwater, USA) which uses chemiluminescent immunoassay (CLIA) technology for the quantitative determination of 25-hydroxyvitamin D in human serum. The measuring range of DiaSorin LIAISON® 25 OH Vitamin D assay was 4.0–150 ng/mL. This analysis was accredited at the performing laboratory: the laboratory of clinical chemistry at the Södra Älvsborg Hospital in Borås, Sweden.

### Statistical analysis

The first objective was to describe the population by number of individuals, age, gender, 25OHD concentrations, vitamin D supplementation, Fitzpatrick skin phototype, average number of hours outdoors per week during April to August, lack of appetite ≥3 months, antibiotics during the last 6 months and having dementia or not.

The second objective was to calculate the prevalence of vitamin D deficiency defined by four different cut-off values: 25OHD < 12.5 nmol/L, 25OHD < 25 nmol/L, 25OHD < 50 nmol/L and 25OHD < 75 nmol/L. This was calculated for all residents and for residents divided by having vitamin D supplementation or not. The prevalence of vitamin D deficiency (25OHD < 25 nmol/L) was compared between residents with or without dementia using Pearson’s chi-square test.

The third objective was to clarify whether previously known factors for vitamin D deficiency were associated with vitamin D deficiency (25OHD < 25 nmol/L), as well as dementia and antibiotic treatments during the last 6 months, in a nursing home population, adjusted for age and gender. Adjusted and unadjusted logistic regressions were performed with vitamin D deficiency (25OHD < 25 nmol/L) as the dependent variable and the following independent variables: age, gender, vitamin D supplementation, Fitzpatrick skin phototype (4–6), number of hours outdoors per week, lack of appetite ≥3 months, dementia and any antibiotic treatments during the last 6 months. Zero order correlations between independent variables were checked and correlations >0.6 were not allowed.

Statistical significance was considered *p* < 0.05. IBM SPSS Statistics version 22 was used for statistical analysis (IBM Corporation, Armonk, New York, United States).

## Results

### Studied population

**Fig. 1:**
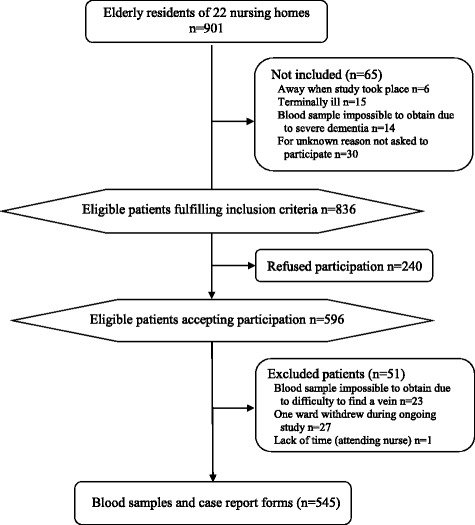
Participant flow chart

Inclusion criteria were fulfilled by 836 of 901 residents in 22 nursing homes, and 596/836 (71%) accepted participation (Fig. 1). Blood samples and case report forms were provided from 545 residents, 370 (68%) women and 175 (32%) men. Mean age was 86 years (SD 6.9), range 56–102. Women (mean 87 years, SD 6.7, range 62–100) were slightly older than men (mean 85 years, SD 7.1, range 56–102) (*p* = 0.0039).

Among participating residents 55% (299/545) had dementia, 30% (164/543) had received any antibiotic treatment during the previous 6 months (this was unknown for 2 participants) and 45% (247/545) had lack of appetite ≥3 months. There was no association between lack of appetite ≥3 months and diabetes (*p* = 0.49), dementia (*p* = 0.55), rheumatic diseases (*p* = 0.15), cancer (p = 0.15) and pressure ulcers (*p* = 0.73). The average number of hours outdoors per week during April to August was known for 489 residents: mean 6.6 h per week (SD 7.1), median 5.0 h per week and range 0–50 h. Twenty percent of the residents spent ≤1.0 h outdoors per week during April to August. Fitzpatrick skin phototype was known for 541 residents, 71% (385/541) had phototype 1–3 and 29% (156/541) had phototype 4–6. Vitamin D supplementation was recorded in 17% (94/545) and of these 85% (80/94) had colecalciferol (vitamin D_3_) and 15% (14/94) had ergocalciferol (vitamin D_2_).

### Serum 25OHD concentrations

Among all residents, regardless of vitamin D supplementation: 3.7% (20/545) had 25OHD < 12.5 nmol/L, 41% (224/545) had 25OHD < 25 nmol/L, 82% (448/545) had 25OHD < 50 nmol/L and 94% (510/545) had 25OHD < 75 nmol/L. The number of residents in between the different cut-off values are presented in Table 1, as well as residents with and without vitamin D supplementation separate.

**Table 1:** Serum 25-hydroxyvitamin D (25OHD) concentrations among nursing home residents

	All residents^a^	Residents without vitamin D supplementation	Residents with vitamin D supplementation
25OHD < 12.5 nmol/L	3.7% (20/545)	4.4% (20/451)	0% (0/94)
25OHD 12.5 to <25 nmol/L	37% (204/545)	44% (198/451)	6.4% (6/94)
25OHD 25 to <50 nmol/L	41% (224/545)	45% (203/451)	22% (21/94)
25OHD 50 to <75 nmol/L	11% (62/545)	4.9% (22/451)	43% (40/94)
25OHD > 75 nmol/L	6.4% (35/545)	1.8% (8/451)	29% (27/94)

^a^In total 545 residents: 451 residents without vitamin D supplementation and 94 residents with vitamin D supplementation

Residents with dementia had lower 25OHD concentrations (mean 31 nmol/L, SD 19) compared to residents without dementia (mean 38 nmol/L, SD 22) (*p* < 0.001). Vitamin D deficiency defined as 25OHD < 25 nmol/L was more common (p < 0.001) among residents with dementia; 51% (151/299) versus 30% (73/246) in residents without dementia.

### Factors associated with vitamin D deficiency

Adjusted OR (95% CI; *p*-value) for possible predictors of vitamin D deficiency (25OHD < 25 nmol/L): vitamin D supplementation 0.075 (0.031–0.18; p < 0.001), lack of appetite ≥3 months 0.75 (0.50–1.1; *p* = 0.15), hours outdoors/week 0.99 (0.96–1.0; *p* = 0.62), Fitzpatrick skin phototype (4–6) 0.69 (0.44–1.1; *p* = 0.12), dementia 2.3 (1.5–3.4; p < 0.001) and antibiotics last 6 months 1.6 (1.1–2.6; *p* < 0.029), adjusted for age and gender (Table 2).

**Table 2:** Factors associated with serum 25-hydroxyvitamin D (25OHD) < 25 nmol/L

	Unadjusted odds ratio^a^(95% CI; *p*-value)	Adjusted odds ratio^b^ (95% CI; p-value)
Age	0.98 (0.96–1.0; *p* = 0.12)	0.98 (0.95–1.0; *p* = 0.22)
Gender^c^	1.1 (0.74–1.5; *p* = 0.72)	1.4 (0.92–2.2; *p* = 0.12)
Vitamin D supplementation	0.073 (0.031–0.17; ***p*** **< 0.001**)	0.075 (0.031–0.18; **p < 0.001**)
Fitzpatrick skin phototype (4–6)^d^	0.54 (0.36–0.80; ***p*** **= 0.0022**)	0.69 (0.44–1.1; p = 0.12)
Hours outdoors/week^e^	0.98 (0.96–1.0; *p* = 0.15)	0.99 (0.96–1.0; *p* = 0.62)
Lack of appetite ≥3 months	0.93 (0.66–1.3; *p* = 0.66)	0.75 (0.50–1.1; p = 0.15)
Dementia	2.4 (1.7–3.4; **p < 0.001**)	2.3 (1.5–3.4; **p < 0.001**)
Antibiotics last 6 months	1.2 (0.85–1.8; *p* = 0.28)	1.6 (1.1–2.6; ***p*** **= 0.029**)

^a^N = 545 for age, gender, vitamin D supplementation, lack of appetite and dementia. *N* = 541 for Fitzpatrick skin phototype. *N* = 489 for hours outdoors/week. *N* = 543 for antibiotics last 6 months

^b^N = 488 included in analysis. Adjusted logistic regressions with 25OHD < 25 nmol/L as the dependent variable and the following independent variables: age, gender, vitamin D supplementation, Fitzpatrick skin phototype, hours outdoors/week, lack of appetite, dementia and antibiotics last 6 months

^c^Reference category: male

^d^Reference category: Fitzpatrick skin phototype 1–3

^e^Average number of hours outdoors/week during April to August

Statistically significant findings are bold

## Discussion

Vitamin D deficiency was common among nursing home residents and strongly associated with dementia. As expected vitamin D supplementation was associated with lower chance of vitamin D deficiency, however appetite, staying outdoors and skin phototype were not significant predictors in the model. Antibiotic treatments during the last 6 months were associated with vitamin D deficiency.

### Strengths and limitations of the study

A strength of this study is that blood samples were collected from every resident accepting participation and of whom it was possible to get a blood sample during three winter months (January to March). However, 240 of the individuals registered at the nursing homes refused participation. We obtained blood samples and study protocols from 60% (545/901) of all individuals registered at the nursing homes, those not fulfilling inclusion criteria are also included in the total number of registered individuals (Fig. 1). This may appear low but it is similar or higher than previously published studies in nursing homes [24, 35, 43]. Still this may be considered acceptable when studying an elderly frail population with a high proportion of residents with dementia as well as the ethical requirement of approval from appointed representative/relatives.

It is a strength that information was collected about appetite, diagnosis of dementia, antibiotic treatments during the last 6 months (as a marker of potential bacterial infection), skin type and the average number of hours outdoors per week during April to August. As the duration of lack of appetite is of importance we have only registered lack of appetite if the duration was ≥3 months. However, it is a weakness that we did not register the total duration of lack of appetite.

There is a lack of consensus regarding the optimum serum concentrations of vitamin D [28, 29], therefore we calculated the prevalence of vitamin D deficiency by four different cut-off values: 25OHD < 12.5 nmol/L, 25OHD < 25 nmol/L, 25OHD < 50 nmol/L and 25OHD < 75 nmol/L. When evaluating factors associated with vitamin D deficiency we used only one cut-off value, 25OHD < 25 nmol/L, representing moderate/severe vitamin D deficiency [31].

According to previous studies vitamin D deficiency might be associated with reduced cognitive functions and dementia [9–14], also supported by our study where 25OHD < 25 nmol/L was strongly associated with dementia (*p* < 0.001, Table 2). However, with this study design we cannot tell whether vitamin D deficiency caused/worsened dementia, or if it is the other way around, that dementia caused vitamin D deficiency. Although dementia diagnosis was the result of clinical findings and investigation it was not standardised and so precise diagnostic criteria will be subject to some variation.

Antibiotic treatments during the last 6 months, as a marker of potential bacterial infection, were associated with vitamin D deficiency (*p* = 0.029, Table 2). This potentially supports the hypothesis that vitamin D deficiency is associated with susceptibility to infections [16–20]. A weakness regarding this result is that the unadjusted OR was not significant (*p* = 0.28, Table 2), therefore the association needs to be interpreted with caution.

Decreased renal function is one factor affecting the vitamin D concentrations, however in this study laboratory tests regarding renal function were not taken [25].

### Comparison with existing literature

Vitamin D deficiency was common among Swedish nursing home residents in this study, as well as in previous studies in other countries [19, 24, 29, 35–43]. As expected vitamin D supplementation predicted less vitamin D deficiency. However appetite, staying outdoors and skin phototype were not significant predictors in the model, which differ from previous studies of younger individuals. Possibly these factors are less important in this population since they stay outdoors only for a few hours a week, likely with clothes covering most of their skin or beneath the sunshade. Also the nutritional status and appetite is generally decreased in this population, which might explain why lack of appetite did not influence the vitamin D concentrations in this study. The results are important in clinical practice as it provides knowledge about vitamin D deficiency, since elderly can have vitamin D deficiency regardless of more time spent outdoors and a relatively good appetite.

There is one recent study regarding vitamin D deficiency among elderly residents of Swedish nursing homes. This study also showed high prevalence of vitamin D deficiency, but only 14% of the 333 participating residents had 25OHD < 25 nmol/L at inclusion [24], compared to 41% in our study. However, the aims and study designs differed between these two studies. As the aim of the previous study was to examine whether low levels of 25OHD were associated to an increased risk of mortality, baseline samples were obtained when residents were moving into the nursing homes. These new residents were expected to have higher serum concentrations of vitamin D compared to residents who have lived at the nursing home for a longer time. The previous study also included patients throughout the years 2007 to 2011 and there were seasonal changes in 25OHD concentrations. In our study all blood samples were taken during January to March in a single year.

### Implications for research and practice

Clinicians have to be aware of the high prevalence of vitamin D deficiency among elderly residents of nursing homes. Vitamin D deficiency was strongly associated with dementia, therefore there is need for future studies to clarify if there is a causal relationship between vitamin D deficiency and dementia. Regardless of causality or not, it is important to be alert for vitamin D deficiency in residents with dementia as half of them had 25OHD < 25 nmol/L. As antibiotic treatments during the last 6 months were associated with vitamin D deficiency further research is important to clarify whether treatment of vitamin D deficiency can decrease the number of potential antibiotic requiring infections.

## Conclusions

Vitamin D deficiency was common among nursing home residents and strongly associated with dementia. Regardless of causality or not, it is important to be alert for vitamin D deficiency in nursing homes residents with dementia. As expected vitamin D supplementation predicted less vitamin D deficiency, however appetite, staying outdoors and skin phototype were not significant predictors in the model. Antibiotic treatments during the last 6 months were associated with vitamin D deficiency, potentially supporting the hypothesis that vitamin D deficiency is associated with susceptibility to infections.

## Abbreviations

25OHD: 25-hydroxyvitamin D
CI: confidence interval
nmol/L: nanomole/l
OR: odds ratio
